# Cell therapy-induced recovery of dysfunctional microvasculature

**DOI:** 10.18632/aging.204183

**Published:** 2022-07-13

**Authors:** Evan Paul Tracy, Gabrielle Rowe, Amanda J. LeBlanc

**Affiliations:** 1 Department of Physiology, University of Louisville, Louisville, KY 40202, USA; 2 Department of Cardiovascular and Thoracic Surgery, University of Louisville, Louisville, KY 40202, USA

**Keywords:** aging, adrenergic, stromal vascular fraction

In aging, circulating catecholamines rise to counter a decline in cardiac output. While initially protective, this can lead to decreased sensitivity of dilative microvascular beta-adrenergic receptors (βAR), increasing risk of ischemia through poor perfusion. Our lab has shown an intravenous injection of adipose stromal vascular fraction (SVF) significantly improves β1AR mediated coronary flow reserve (CFR) and restores the vasodilatory capacity of coronary vessels to β1AR agonists in aged female rats [1]. Our recent study in the journal Geroscience examined alterations in the signaling pathway of the β1AR in response to aging that induced desensitization and are improved or unchanged due to SVF therapy [2]. The objective of this editorial is to address additional points of consideration and suggest new avenues worth investigating.

In aging female rats, there was an increase in plasma norepinephrine (NE) concentration that was reduced following SVF therapy (albeit not significant) [1]. However, when measuring urine catecholamines SVF did not reduce the significantly increased level of NE present in aging [2]. There was a significant reduction in transcription of catecholamine degradation enzyme catechol-o-methyltransferase (COMT, also degrades estrogens) following SVF therapy compared to aged-controls [2]. Importantly, aged-controls had both high NE concentration and higher (relative to SVF) COMT expression, while SVF-injected rats only exhibited high NE concentration (relative to aging), suggesting perhaps that untreated aged-controls are producing enough NE to overcome NE degradation by the enhanced presence of degradation enzymes. This data could indicate less total catecholamine production after SVF therapy which could have an impact on β1AR functionality and desensitization.

Transcription of the β1AR is not varied in aging or after SVF therapy (albeit β2AR transcription was increased by SVF vs. aging) [2]. Therefore, we investigated the downstream signaling cascade and regulation of the β1AR. GRK2 phosphorylates the β1AR following catecholamine binding; an initial step in receptor internalization. While protein expression of GRK2 was unchanged between groups, there was an aging-associated increase in naturally active GRK2 compared to phosphorylated, naturally inactive, GRK2 (partially reversed by SVF). We showed that inhibition of GRK2’s kinase activity improved β1AR vasodilatory sensitivity in coronary microvessels of aged and SVF-treated rats.

It’s important to examine the contribution of nitric oxide (NO), considering its depletion in aging and its ability to inhibit GRK2-mediated β1AR internalization. Since reactive oxygen species (ROS) can scavenge NO, we also examined the ROS-dependent effects on β1AR desensitization and internalization. Coronary microvessels from aged rats were shown to have increased ROS and reduced bioavailability of NO [3]. Exogenous ROS incubation impairs young control β1AR mediated dilation (mimicking aging) while exogenous low-dose sodium nitroprusside (NO donor) restores old control β1AR mediated dilation (mimicking youth) [4]. Plentiful NO (such as during youth) naturally inhibits GRK2s by nitrosative post-translational modification. With SVF therapy, although NO bioavailability is not restored, ROS levels are reduced possibly alleviating β1AR desensitization and internalization [3,4].

SVF therapy may promote less impeded recycling with reduced degradation relative to aging. GRK2 recruits beta-arrestins to form clathrin/dynamin coated vesicles for receptor endocytosis into endosomes. PP2A dephosphorylates the β1AR allowing for recycling back to the plasma membrane. Alpha-arrestin is responsible for ubiquitination-mediated internalization of βARs as well as to delay their recycling [5,6]. Of relevance, SVF significantly decreased alpha-arrestin 3 transcription compared to aged controls in isolated coronary microvessels [2].

SVF therapy also increased transcription of the estrogen related receptor-α and G-protein coupled estrogen receptor 1 (Gper1) compared to old control. Estrogen induces vasorelaxation through NO-dependent mechanisms in response to direct agonism or flow, and estrogen receptor alpha activation attenuates oxidative stress [7]. Additionally, estrogen and estrogen receptor-β are functionally linked to βAR receptor transcription and dilative response to norepinephrine [8]. Females of advanced age exhibit a drop in circulating estrogen levels, impairing microvascular function. Elevated estrogen receptor levels in SVF-treated rats may make their microvessels more sensitive to the limited circulating estrogen in aging, which could have regenerative effects in the context of enhanced β1AR function after SVF-therapy.

Taken together our data suggests that aging results in β1AR dysfunction through catecholamine overdrive, enhanced active (dephosphorylated) GRK2, accumulation of ROS, and loss of NO-mediated inhibition of GRK2 nitrosylation, all of which culminate in internalization of the β1AR. Possible explanations for SVF-mediated recovery of coronary microvascular β1AR vasodilatory capacity could be through a more homeostatic catecholamine microenvironment, reducing ROS accumulation and its mediation of desensitization and internalization, and possibly by improving β1AR receptor recycling or reducing ubiquitination by reducing alpha arrestin 3 expression.

**Figure 1:**
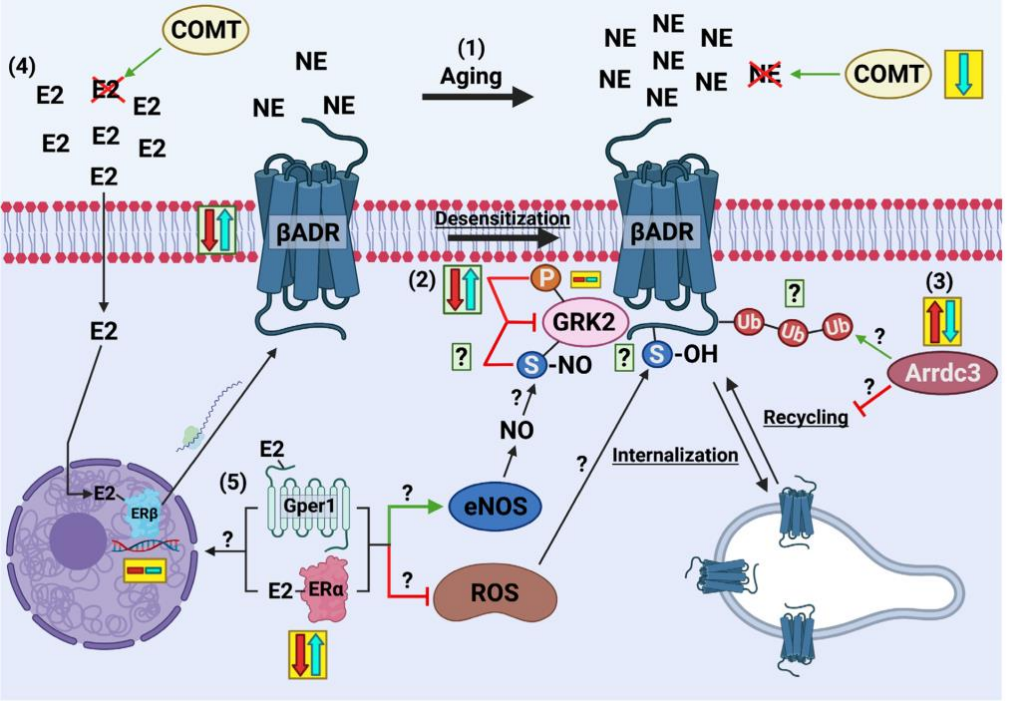
**Potential contributing mechanisms of SVF-induced reversal of aging-mediated βADR dysfunction.** In aging, there is an overdrive of catecholamine production that facilitates βADR desensitization and internalization mediated by GRK2 (1). Transcription of catecholamine degradation enzyme COMT is significantly reduced by SVF therapy. There is no change in aging or SVF therapy in GRK2 transcription, however, naturally inhibited GRK2 (phosphorylated) is decreased in aging partially restored by SVF (albeit non-significantly) (2). Whether other post-translational modifications such as inhibitory nitric oxide-mediated S-nitrosylation of GRK2 contribute to SVF-mediated recovery of βADR function warrants investigation. The transcription of alpha arrestin 3 (arrdc3) was significantly enhanced in aging and reversed by SVF (3). Whether this contributes to coronary microvascular βADR dysfunction in aging, as it does in other vascular settings through βADR ubiquitination and delayed recycling, remains unknown. In other vascular settings, estrogen enhances βADR vasodilatory function and transcription via estrogen receptor-β, although there were no transcriptional differences between groups in our study (4). Transcription of estrogen receptor-α and Gper1 were significantly enhanced with SVF therapy, which may influence βADR transcription or enhance nitric oxide or attenuate ROS production as they are known to do in other vascular settings, which could influence βADR dilatory function (5). Yellow boxes represent gene expression whereas green boxes represent protein expression with red arrow indicating aging and blue arrow indicating SVF therapy. Question marks represent future directions to elucidate SVF-mediated recovery of βADR function based on RNA sequencing data. Image created with BioRender.com.

Future study is required to fully elucidate the mechanisms by which aging and SVF therapy modulate β1AR desensitization, internalization, and recycling (Figure 1). This is especially poignant in the context of regulatory post-translational modifications (thiol oxidation, nitrosylation, ubiquitination, phosphorylation) in part through ROS and NO, which fluctuate with aging and SVF therapy [3]. Protein expression and functional significance of alpha arrestin 3 on recycling and ubiquitination, as well as the significance of estrogen receptor alpha and Gper1 in the perseverance of β1AR function are especially pertinent and require further investigation. Understanding the intricate mechanistic underpinnings of coronary microvascular dysfunction is of great importance considering first line therapies include beta blockers, which could potentiate the root cause of pathology despite providing symptomatic management.
